# Development of Orally Active Thrombin Inhibitors for the Treatment of Thrombotic Disorder Diseases

**DOI:** 10.3390/molecules200611046

**Published:** 2015-06-15

**Authors:** Li-Wei He, Wei-Chen Dai, Nian-Guang Li

**Affiliations:** Department of Medicinal Chemistry, Nanjing University of Chinese Medicine, Nanjing 210023, China; E-Mail: heliwei@njutcm.edu.cn

**Keywords:** thrombin, thrombotic disorders, allosteric inhibitors, natural products, orally active, coagulation, thrombosis

## Abstract

Thrombotic disorders represent the major share of the various cardiovascular diseases, and significant progress has been made in the development of synthetic thrombin inhibitors as new anticoagulants. In addition to the development of highly potent and selective inhibitors with improved safety and suitable half-life, several allosteric inhibitors have been designed and synthesized, that did not fully nullify the procoagulant signal and thus could result in reduced bleeding complications. Furthermore, natural products with thrombin inhibitory activity have been isolated, and some natural products have been modified in order to improve their inhibitory activity and metabolic stability. This review summarizes the development of orally active thrombin inhibitors for the treatment of thrombotic disorder diseases, which could serve as a reference for the interested researchers.

## 1. Introduction

Thrombotic disorders, which are resulted from abnormalities in the blood flow, coagulation cascade or fibrinolysis, can lead to deep vein thrombosis, myocardial infarctions and strokes. These cardiovascular disorders represent the major share of the various cardiovascular diseases in the World [1]. Although warfarin is an oral antithrombotic agent, it possesses a number of limitations [2] including an indirect action mechanism, the need for constant monitoring to assure effective drug plasma levels and avoidance of bleeding complications, and potential drug–drug interactions. Other antithrombotics such as heparin and low-molecular-weight heparin, must be given parenterally because they lack oral bioavailability, which is a major obstacle for the chronic treatment of thromboembolic diseases [3]. Although coumarin analogs demonstrate oral activity, careful monitoring of treated patients is necessary in order to avoid their side effects [4].

**Figure 1:**
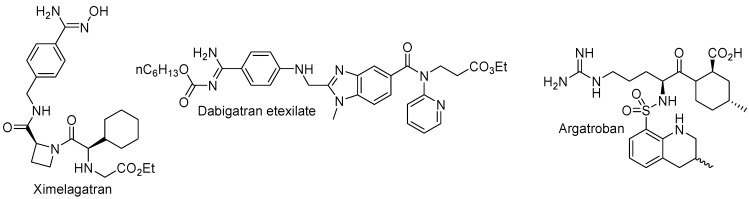
The chemical structures of three small molecule thrombin inhibitors.

Being a blood coagulation enzyme, thrombin plays a critical role in hemostasis and thrombosis [5]. Therefore, thrombin inhibitors have long been recognized as potential therapeutic agents for the treatment of a variety of thromboembolic disorders, including deep vein thrombosis, pulmonary embolism, atrial fibrillation and thromboembolic stroke. Three small molecule thrombin inhibitors have reached the market, Ximelagatran (Exanta, Astra Zeneca, Figure 1), as a prodrug of melagatran, was the first orally direct thrombin inhibitor introduced into the market, however it was withdrawn from the market in 2006 due to hepatotoxicity [6]. Two newer direct thrombin inhibitors, dabigatran etexilate (Pradaxa^®^, Boehringer Ingelheim, Figure 1) and argatroban (GSK, Figure 1), are currently used on the market [7].

During the past three decades, significant progress has been made, in the development of synthetic thrombin inhibitors as new anticoagulants. Straub *et al.* summarized the research on thrombin inhibitors with 26 references in 2011 [8]. Mehta *et al.* have published an update on recent (2010–2013) patents on thrombin inhibitors [9]. Steinmetzer and Stürzebecher summarized the synthetic thrombin inhibitors with both poor and high selectivity in 2004 [10]. Das and Kimball gave a brief review of indirect and direct thrombin inhibitors in 1995 [11]. This review focuses on the development of orally active thrombin inhibitors for treating thrombotic disorders since 2010, and thus could serve as an updated reference for interested researchers.

## 2. Orally Active Thrombin Inhibitors

### 2.1. Selective and Orally Active Thrombin Inhibitors

**Figure 2:**
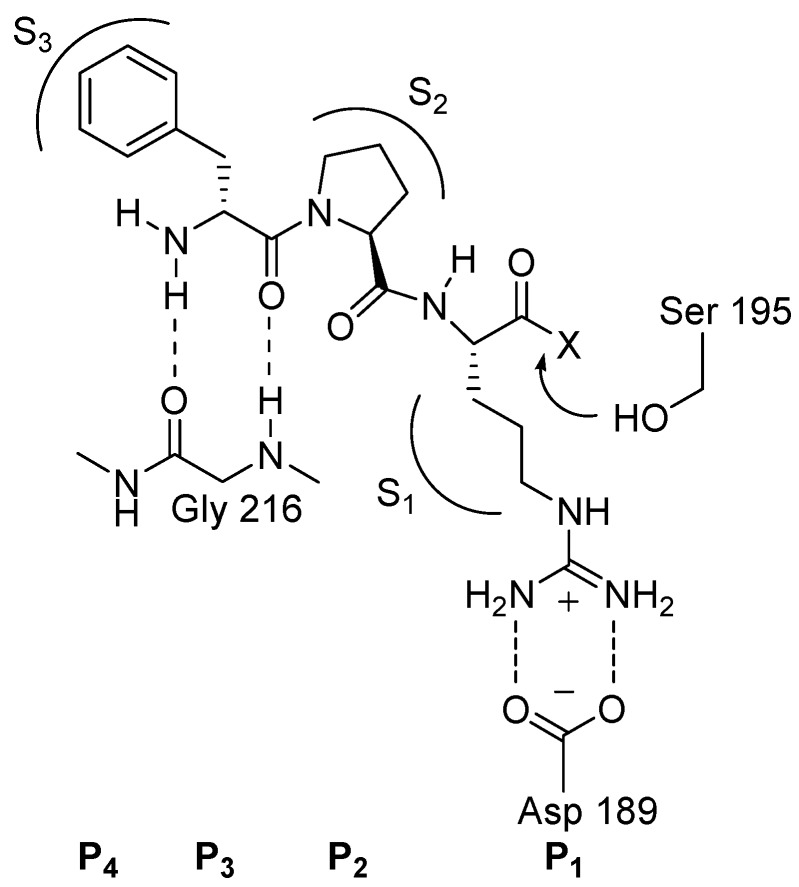
Schematic representation of the binding mode of a d-Phe-Pro-Arg type inhibitor to the active site of thrombin.

Structurally, thrombin’s active site [12] (Figure 2) is composed of a catalytic triad (Ser195, His57, and Asp102), a basic-recognition S1 pocket (Asp189 is the most important residue), a hydrophobic proximal S2 pocket (Tyr60A and Trp60D are the main residues) and a bigger lipohilic distal S3 pocket (Leu 99, Ile174, and Trp215) [10]. Additionally, subsites of S1′, S2′, S3′ and the oxyanion hole have also been considered in the design of stronger and more selective thrombin inhibitors [13].

In order to discovery selective and direct thrombin inhibitors possessing oral bioavailability, the effort started with a tripeptide template, d-Phe-Pro-Arg-H [14]. This fragment interacts with three essential binding sites on the thrombin enzyme (Figure 2), which include the S1 specific pocket and two hydrophobic pockets, the proximal S2 and distal S3. Asp 189 in the S1 specific pocket forms a salt bridge with the guanidine functionality, in order to take advantage of this interaction, many initial inhibitors usually contained a guanidine or other highly basic groups, such as benzamidine and imidazole. Although this strategy often provided increased potency, a concomitant decrease in oral bioavailability and poor pharmacokinetic (PK) properties was noted. In order to improve the PK properties of the drug molecules [15], non-peptide inhibitors have been designed and synthesized.

**Figure 3:**
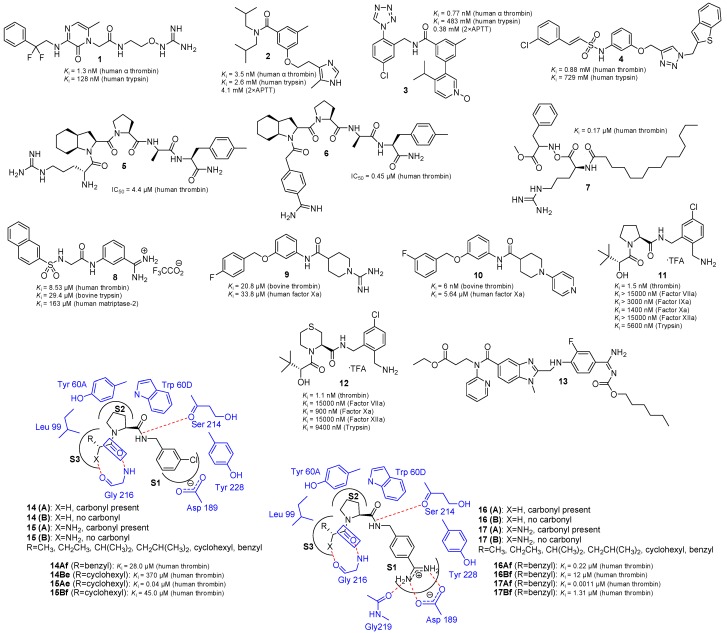
The chemical structures of selective thrombin inhibitors.

In 2010, Lu *et al.* [16] combined the pyrazinone core and the oxyguanidine moiety [17] in one molecule. The oxyguanidine possessed a greatly reduced pKa of 7.0–7.5 which was lower than that of guanidine at 13–14, thus facilitating favorable *in vitro* permeability and PK properties. Thus, RWJ-671818 (**1**, Figure 3) was identified as a novel, orally active and selective human α-thrombin (*K*_i_ = 1.3 nM) inhibitor (*K*_i_ = 128 nM for human trypsin, about 100-fold), which was potentially useful for the acute and chronic treatment of venous and arterial thrombosis. In a rat deep venous thrombosis model, oral administration of **1** at 30 and 50 mg/kg reduced the thrombus weight by 87% and 94%, respectively. Furthermore, this compound showed excellent oral bioavailability of 100% in dogs with an estimated half-life of approximately 3 h. On the basis of this noteworthy preclinical data, **1** was advanced into human clinical trials, and successfully progressed through phase 1 studies.

Structure-based design techniques [18] generated a new lead inhibitor **2** (Figure 3). In 2011, SAR work by Isaacs’s group [19] at both the amide and ether carbons of the central benzene ring, in conjunction with optimization of the P1 ligand, led to the development of a new series of potent inhibitors exemplified by compound **3** (Figure 3), which was obtained by increasing the polarity via oxidation to the pyridine N-oxide. This compound selectively inhibited the thrombin with its *K*_i_ value was 0.77 nm (*K*_i_ = 483 mM for trypsin, about 627 million-fold), furthermore, its activity in the 2 × APTT assay (2 × APTT = 0.38 μM) was also significantly improved.

In 2011, Freire’s group [20] synthesized 25 triazole/tetrazole-based sulfonamides and evaluated them against thrombin, trypsin, tryptase and chymase, and found that the triazole-based sulfonamides inhibited thrombin more efficiently than the tetrazole counterparts. Particularly, compound **4** (Figure 3) showed strong thrombin inhibition activity (*K*_i_ = 880 nM), and significant selectivity against other human related serine proteases like trypsin (*K*_i_ = 729 μM, about 800-fold). Furthermore, it was demonstrated that the thrombin binding affinity of this new triazole-based scaffold was enthalpically driven, thus making it a good candidate for further development.

Previous SAR studies [21] on the bradykinin breakdown product Arg-Pro-Pro-Gly-Phe led to the lead compound **5** (d-Arg-Oic-Pro-d-Ala-Phe(*p*-Me)-NH_2_) (Figure 3). The X-ray structure of **5** in the thrombin active site revealed which sites could be modified to potentially improve the inhibitory activity. In 2011, Mosberg’s group [22] synthesized nine peptides in which only the d-Arg residue in **5** sequence was replaced, thus investigating some effects including conformational restriction, modification of the basic moiety at the end of the side chain, and removal of the charge from the *N*-terminus that could affect the biological activity. Compound **6** (IC_50_ value of 0.45 μM, Figure 3) showed similar potency to **5**.

In 2012, Poyarkov *et al.* [23] employed the d-Arg-d-Phe motif to create new thrombin inhibitors which were resistant to enzymatic degradation. Previously they had synthesized peptide-based inhibitors resistant to enzymatic degradation, such as Chrom-d-Arg-d-Phe-OMe and Laur-d-Arg-d-Phe-OMe, where Chrom was a 3-[6-ethyl-7-hydroxy-3-(4-methylthiazol-2-yl)-4-oxo-4*H*-chromen-2-yl]-propionic acid residue and Laur was a lauric acid residue [24]. The analysis of the thrombin inhibitory activity demonstrated that the modification of the fatty acid residue appeared to be the most successful one, and introduction of the lauric acid residue maximally increased the inhibition effect, so they developed the following novel inhibitors: Myr-d-Arg-d-Phe-OMe, where Myr was a myristic acid residue, and Fmoc-d-Arg-d-Phe-OMe, where Fmoc was a 9-fluorenylmethoxycarbonyl group. This modification afforded the most potent thrombin inhibitor **7** (Figure 3), with dramatically increased inhibition efficacy (*K*_i_ = 0.17 μM) and selectivity toward factor X, plasmin and trypsin (more than 600, 900, and 5000-fold, respectively).

Because of their arginine mimicking properties, benzamidine substructures [25] have been extensively utilized in the rational design of inhibitors for trypsin-like serine proteases. A binding mode study for benzamidine inhibitors and trypsin-like serine proteases showed that the benzamidine group was deeply buried in the S1 specific pocket, where a negatively charged conserved Asp189 at the bottom was engaged in a salt bridge with the positively charged benzamidine functionality [26]. Consequently, in 2012, Dosa *et al.* [27] synthesized some compounds which contained a fixed sulfamoyl benzamidine moiety for accommodation in the S1 pocket, and several linker-connected functionalities for additional interactions with the S3/S4 binding region. Then they evaluated them against three prototype serine proteases including bovine trypsin, human thrombin and human matriptase-2. They found that compound **8** (Figure 3) was a potent human thrombin inhibitor with its *K*_i_ value was 8.53 µM, it could selectively inhibit bovine trypsin, and human matriptase-2, with the *K*_i_ values were 29.4 µM (3.4-fold) and 163 µM (19.1-fold) respectively.

In 2013, de Candia *et al.* [28] designed and synthesized a new class of non-peptide direct thrombin inhibitors, built on the 1-(pyridin-4-yl)piperidine-4-carboxamide structure. Starting from a strongly basic 1-amidinopiperidine derivative **9** (Figure 3) which had poor thrombin (*K*_i_ = 20.8 μM) and factor Xa (*K*_i_ = 33.8 μM) inhibitory activities, they considerably improved the anticoagulant activity and artificial membrane permeability, by optimizing the basic P1 and the X-substituted phenyl P4 binding moieties. SAR studies led to compound **10** (Figure 3), which showed excellent thrombin inhibitory activity (*K*_i_ = 6 nM), weak anti-factor Xa activity (*K*_i_ = 5.64 μM, 940-fold), and remarkable selectivity over other serine proteases. Compound **10** also showed *in vitro* anticoagulant activity in the low micromolar range and significant membrane permeability. In mice (*ex vivo*) at 2 h after oral dosing (100 mg·kg^−1^), **10** demonstrated anticoagulant effects with a significant 43% prolongation of the activated partial thromboplastin time (aPTT).

Pyrrolidine **11** (Figure 3) was a potent thrombin inhibitor discovered [29] at Merck several years ago. In their original report, Morissette *et al.* noted that reducing the core ring size (*i.e*., replacement of pyrrolidine with azetidine) resulted in reduced thrombin inhibitory activity. However, the effect of ring expansion was unknown. In an attempt to discover an improved thrombin inhibitor, and to more fully elucidate the SAR of the heterocyclic core of **11**, in 2014, Blizzard *et al.* [7] prepared a series of six-membered heterocyclic core analogs of **11**, in which the pyrrolidine core was replaced by various heterocycles, and evaluated them against thrombin, clotting factors VIIa, IXa, Xa, XIIa, and trypsin. Interestingly, the thiomorpholine analog **12** (Figure 3) was the most active one among the seven analogs, exhibiting thrombin inhibitory activity (*K*_i_ = 1.1 nM) comparable to that of the pyrrolidine **11** (*K*_i_ = 1.5 nM), and selectivity toward factor VIIa, Xa, XIIa and trypsin (more than 13,600, 800, 13,600, and 8500-fold, respectively). Unfortunately, in a dog PK study (IV dosing), thiomorpholine **12** exhibited poorer PK than the lead pyrrolidine **11**, this was consistent with the reduced hepatocyte stability of **12** (76% remaining at *t* = 90 m) *vs.* **11** (93% remaining) in dog. None of the analogs inhibited CYP 3A4, CYP 2D6, or CYP 2C9 at concentrations up to 50 µM. Due to its poor PK relative to pyrrolidine **11**, thiomorpholine **12** were not evaluated in the further study.

Dabigatran may have a number of advantages, but at only 6.5% its bioavailability is low [30]. Furthermore, one major disadvantage was a dose-dependent risk of haemorrhage [31]. To minimize this side effect and solve the problem of low bioavailability, new dabigatran derivatives have been developed. Hauel [32] found that benzimidazole could closely combine with the thrombin active site to exert anticoagulant activity, and bind to a branch of amidinophenylalanine as a false arginine [33]. Furthermore, benzamidine interacted closely with the residues in the thrombin active site cleft, and then formed a two-tooth salt bridge with the carboxylic ester of Asp189. By contrast, the pyridine ring of dabigatran was not necessary for its bioactivity. Based on these previous studies, in 2015, Li *et al.* [34] synthesized 21 new fluorinated derivatives with bioisosteric replacement using dabigatran as a basic skeleton. Compound **13** (Figure 3) showed a fairly strong inhibitory activity on thrombin-induced platelet aggregation, furthermore, in a rat arteriovenous thrombosis model, **13** demonstrated potent activity with an inhibition rate of (73 ± 6)%, which was comparable to that of dabigatran etexilate (76 ± 2)%.

In order to reveal new aspects of ligand functional group’s cooperative contributions to the binding free energy, in 2015, Said *et al.* [35] designed and synthesized four series of structurally related thrombin inhibitors. As shown in Figure 3, these scaffolds included a proline which bound in the S2 pocket, fitting under the Tyr60A and Trp60D of the 60-loop (similar to the natural substrate). The difference between the two categories of inhibitors was that **14** and **15** (Figure 3) had a *m*-chlorobenzyl moiety binding in the S1 pocket, whereas **16** and **17** (Figure 3) had a more firmly bound (salt bridge and three H-bonds) benzamidine moiety. The *K*_i_ values for the most potent compounds in series of **14**–**17** were provided in Figure 3. Within each of these categories, the size of the P3 hydrophobic side chain was gradually increased from a methyl to a benzyl side chain, in the presence or absence of the carbonyl group (blue box) that was H-bonded to the amino group of Gly216 residue. In order to investigate the effect of the presence or absence of an additional adjacent hydrogen bonding moiety, on the cooperativity between the carbonyl group and the hydrophobic side chain, analogous ligands wherein X = H or NH_2_ (H-bonds with the protein carbonyl oxygen of the Gly216 residue) were also prepared and analyzed. Using four different series of thrombin inhibitors, Said *et al.* revealed a strong positive cooperativity between a H-bond accepting carbonyl functionality and the adjacent P3 hydrophobic side chain. Adding an H-bond donating amine adjacent to the P3 hydrophobic side chain further increased this positive cooperativity, thereby improving the *K*_i_ by 546-fold. In contrast, adding an amidine multiple H-bond/salt bridge group in the distal S1 pocket did not affect this cooperativity. An analysis of the crystallographic B-factors of the ligand groups inside the binding site, indicated that the strong cooperativity was mainly due to a significant mutual reduction in the residual mobility of the hydrophobic side chain, and the H-bonding functionalities that was absent when the separation distance was large. This type of cooperativity was important to encode in binding affinity prediction software, and to consider in SAR studies.

### 2.2. Allosteric Thrombin Inhibitors

Allosteric regulation is a promising strategy for thrombin inhibition. Nature tends to utilize allosterism to confer recognition specificity and also to affect regulation. For thrombin, the possibility of regulation, or controlled inhibition, was of considerable importance because nearly all current anticoagulants are associated with risk of bleeding. An appropriately designed allosteric regulator that did not fully nullify the procoagulant signal, might maintain a finely tuned balance between procoagulant and anticoagulant signals, thus resulting in reduced bleeding complications.

An exciting alternative to competitive inhibition of thrombin was allosteric inhibition through either exosite I or exosite II. It was well established that binding of ligands in these exosites, could induce conformational changes in the active site of thrombin [36]. An example of this was thrombomodulin, which interacted with exosite I to change the substrate specificity of thrombin from fibrinogen to protein C [37]. Likewise, hirugen binding in exosite I increased or decreased the catalytic efficiency of thrombin, depending on the nature of the chromogenic substrate.

Exosite II ligands included heparin, chondroitin sulfate, haemadin, and fibrinogen γ'. These could be broadly classified as either highly anionic polysaccharides or traditional peptides. The interaction of heparin with exosite II could induce great reactivity with antithrombin, and recent work showed that formation of antithrombin-thrombin complex disrupted exosite II [38].

**Figure 4:**
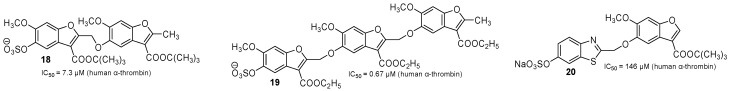
The chemical structures of allosteric thrombin inhibitors.

In pursuit of such allosteric regulators, Desai’s group had earlier designed sulfated low molecular weight lignins (LMWLs) as functional mimetics of heparin which targeted to exosite II-like regions [39,40] of coagulation enzymes, including thrombin [41]. Despite their excellent *in vitro* and *in vivo* anticoagulant properties, sulfated LMWLs were challenging because of their polydispersity and microheterogeneity, which paralleled that observed with the heparins. In 2011, they designed and synthesized 28 monomeric and dimeric sulfated benzofurans [42] derived from the sulfated LMWL structure, as thrombin inhibitors. SAR analysis indicated that sulfation at the 5-position of the benzofuran scaffold was essential for targeting thrombin, Michaelis-Menten kinetic studies showed the inhibition arised from an allosteric process. Plasma clotting assays indicated that the sulfated benzofurans prolonged both the activated partial thromboplastin time and prothrombin time. The *tert*-butyl derivative **18** (Figure 4) was found to be the most potent thrombin inhibitor, with an IC_50_ of 7.3 μM under physiologically relevant conditions.

This work put forward sulfated benzofurans as the first small, synthetic molecules as powerful lead compounds for the design of a new class of thrombin allosteric inhibitors. In order to identify the binding site of sulfated benzofuran dimers (SBDs), in 2012, Desai’s group [43] studied the thrombin inhibitory activity in the presence of exosite 1 and 2 ligands, whereas hirudin peptide and heparin octasaccharide did not affect the thrombin IC_50_ by a high affinity SBD, the presence of full-length heparin reduced inhibition potency by 4-fold. The presence of γ′ fibrinogen peptide, which recognized Arg93, Arg97, Arg173, Arg175, and other residues, resulted in a loss of affinity that correlated with the ideal Dixon−Webb competitive profile. Replacement of several arginines and lysines of exosite 2 with alanine did not affect thrombin inhibition potency, except for Arg173, which displayed a 22-fold reduction in IC50 value. Docking studies suggested a hydrophobic patch around Arg173 was a SBD plausible site which bound to thrombin. This work presented the localization of the SBD binding site, which could lead to thrombin allosteric modulators that were completely different from all clinically used anticoagulants.

In 2013, Desai’s group synthesized monosulfated benzofuran tri and tetrameric homologues [44] of the parent designed dimers. The trimer **19** (Figure 4) was found to be the most potent inhibitor with an IC_50_ value of 670 nM and an efficacy of approximately 80%. Competitive studies using a hirudin peptide (exosite 1 ligand) and unfractionated heparin, heparin octasaccharide, and γ′-fibrinogen peptide (exosite 2 ligands) demonstrated that the exosite 2 recognition was different from that of the parent dimers. Alanine scanning mutagenesis of 12 Arg/Lys residues of exosite 2 revealed the potency in **19** defected for Arg233Ala thrombin, thus confirmed the major difference in recognition site between the two structurally related sulfated benzofurans. These results suggested that multiple avenues were available within exosite 2 for inducing thrombin inhibition.

In order to further design thrombin allosteric inhibitors, in 2014, Desai’s group synthesized a small library of mono sulfated indole and benzothiazole [45] based molecules, and screened them against the panel of coagulation proteases. Compound **20** (Figure 4) was found to have an allosteric mode of inhibition against thrombin (IC_50_ = 146 µM). Unfortunately, no allosteric inhibitor has been shown to be orally active.

### 2.3. Thrombin Inhibitor in Multi-Target Drugs

**Figure 5:**
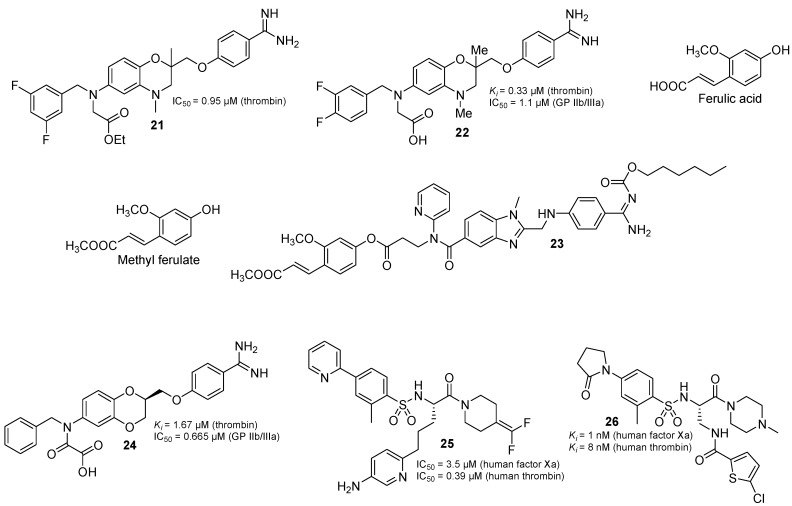
The chemical structures of thrombin inhibitor in multi-target drugs.

Under normal circumstances, endothelial cells played a critical role in preventing intravascular thrombosis: (i) by providing a unique surface which prevented activation of coagulation and platelet aggregation and (ii) by releasing mediators that inhibited prothrombotic processes. However, these endothelial cells were vulnerable to oxidative stress, which was initiated by reactive oxygen species (ROS) diffusing from leukocytes or generated in the endothelium in the context of inflammation [46]. It could therefore be anticipated that antithrombotic therapy with anticoagulant and/or antiplatelet agents, combined with interventions directed against vascular oxidative stress and/or inflammation, both boosting endothelial antithrombotic activity, would display a synergistic action in the treatment of thrombosis. In 2011, Ilić *et al.* synthesized some compounds that combine the thrombin inhibitory activity, lipid peroxidation and lipoxygenase inhibition in the same molecule [47]. The resulting compound **21** (Figure 5) was found to be submicromolar thrombin inhibitor (IC_50_ = 0.95 µM), it was also very potent to inhibit lipid peroxidation (IC_50_ = 36 µM), this made compound **21** an interesting candidate for further investigations towards multi-target antithrombotic drugs.

In order to study the effect of fluorine substituent(s) on anticoagulant and GP IIb/IIIa antagonistic activity, in 2012, Ilić *et al.* [48] synthesized dual antithrombotic compounds based on a 1,4-benzoxazine scaffold by preparing their 3-fluorobenzyl, 4-fluorobenzyl, 3,4-difluorobenzyl and 3,5-difluorobenzyl analogues, bearing a substituent on the position 6 or 7 of the 1,4-benzoxazine scaffold, and found compound **22** (Figure 5) was the most potent compound with balanced dual inhibitory activity (*K*_i(Thr)_ = 0.33 ± 0.07 µM, IC_50(GP IIb/IIIa)_ = 1.1 ± 0.6 µM).

Dabigatran etexilate (Figure 1) is an oral prodrug of dabigatran that could be hydrolysed to generate dabigatran after oral administration. *In vivo*, dabigatran (*K*_i_ = 4.5 nM) could bind to human thrombin selectively, and reversibly to realize a strong and long-lasting anticoagulant effect [32]. Ferulic acid (Figure 5) is one of the components of asafoetida, the dried latex from the giant fennel (*Ferula communis*), which has long been reported to have inhibitory effects on platelet aggregation [49]. In order to combine both the therapeutic benefits of dabigatran and ferulic acid, in 2013, Yang *et al.* coupled dabigatran with methyl ferulate (Figure 5) to afford a series of mutual prodrugs [50]. They expected that the ester bonds of dabigatran-methyl ferulate could be hydrolyzed *in vivo* by esterase to release dabigatran and ferulic acid, which exerted a synergistic inhibitory effect on venous or arterial thrombosis, via two routes—antiplatelet and anticoagulant. The *in vivo* experiments showed that one of the target compounds, **23** (Figure 5, ED_50_ = 3.7 ± 1.0 µmol/kg) possessed more potent activity for inhibiting venous thrombosis, than dabigatran etexilate (ED_50_ = 7.8 ± 1.5 µmol/kg) on the rat venous thrombosis model (Reyers).

Since the combined use of thrombin inhibitors and glycoprotein IIb/IIIa antagonists in the prevention of cardiovascular diseases has shown additional benefits over treatment directed against thrombin or against platelets alone [51], in 2013, Ilić *et al.* reported a series of enantiomeric 1,4-benzodioxine compounds [52] that combine, in the same molecule, highly overlapping thrombin inhibitor and fibrinogen receptor antagonist pharmacophores (Figure 1). The influence of chirality and substitution pattern on thrombin inhibition, and on inhibition of fibrinogen binding to GPIIb/IIIa was analyzed. Docking studies were used to rationalize the results. The (*S*)-isomers of both 2,3-dihydro-1,4-benzodioxine regioisomers at positions 6 and 7 were found to be stronger thrombin inhibitors than the corresponding (*R*)-enantiomers, whereas they observed that stereochemistry did not display a consistent influence on fibrinogen GPIIb/IIIa binding inhibitory activity. Compound **24** (Figure 5), the (*S*)-isomer of the 6-substituted regioisomer, possessed the best balanced dual inhibitory activity, with *K*_i(thrombin)_ = 1.67 ± 0.27 µM and IC_50(GPIIb/IIIa)_ = 0.665 ± 0.26 µM, raising the hope that merging anticoagulant and platelet antiaggregatory activities in the same molecule, could lead to successful multi-target antithrombotic agents.

Starting from compound **25** (Figure 5), with low factor Xa and modest anti-thrombin inhibitory activities (IC_50_’s of 3.5 and 0.39 μM, respectively), in 2013, Meneyrol *et al.* considerably improved the both inhibitory activities [53] through the incorporation of a neutral chlorothiophene P1 fragment, and tuning of P2 and P3–P4 fragments. Final optimization of metabolic stability with microsomes led to the identification of **26** (Figure 5), which displayed strong inhibitory activity *in vitro* vs factor Xa and thrombin (with *K*_i_’s of 1 and 8 nM, respectively). In addition, **26** presented good selectivity *versus* related serine proteases (roughly 300-fold), including trypsin (1000-fold), and was very active (0.39 μM) in the thrombin generation time coagulation assay in human platelet rich plasma. The *in vivo* potent activity in a rat model of venous thrombosis following iv and, more importantly, po administration was also observed (ED_50_ of 0.07 and 2.8 mg/kg, respectively).

### 2.4. Natural Products with Thrombin Inhibitory Activity

**Figure 6:**
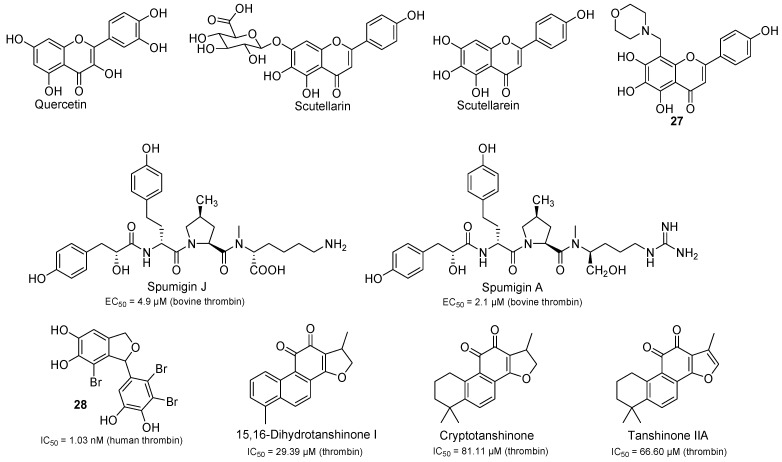
The chemical structures of natural products and their derivatives as thrombin inhibitory activity.

In China, due to an increasing public interest in alternative medicine and disease prevention, traditional Chinese medicines containing rich flavonoids such as *Carthamus tinctorius* L. [54], *Abelmoschus manihot* L. [55], and *Ginkgo biloba* L. [56] have been used in clinic to treat thrombotic diseases for many years, so natural products like flavonoids (e.g., flavones and flavanones) maybe promising lead compounds for thrombin inhibitors. In 2010, we studied the thrombin inhibitory activities of 30 natural flavonoids [57] including flavonols, flavones, flavanones, catechins, anthocyanidins, isoflavones, dihydroflavonols and chalcones, and we found that quercetin (Figure 6) was the strongest thrombin inhibitor among the tested compounds. In blood, quercetin was mainly found in metabolized forms. In order to study the biological activities of these quercetin metabolites in cardiovascular disease, in 2012, we synthesized 17 methylquercetin derivatives [58] based on the *in vivo* metabolism, their thrombin inhibitory activities were evaluated through the analyzation of prothrombin time (PT), activated partial thromboplastin time (APTT), thrombin time (TT) and fibrinogen (FIB). The results showed that six methylquercetin derivatives had stronger inhibitory activities than that of quercetin. Preliminary SARs analysis showed that hydroxyl groups at C-3′ and C-4′ position in the B-ring, and hydroxyl group at C-3 position in the C-ring played key roles in the thrombin inhibitory activity. This finding would provide information for the exploitation and utilization of quercetin as thrombin inhibitor for the treatment of thrombotic disease.

Scutellarin (Figure 6), the major constituent in breviscapine extracted from Chinese herb of *Erigeron breviscapus* (vant.) Hand.-Mazz., showed effectiveness in dilating blood vessels, improving microcirculation, increasing cerebral blood flow, and inhibiting platelet aggregation [59]. It has been clinically used to treat acute cerebral infarction, and paralysis induced by cerebrovascular diseases such as hypertension, cerebral thrombosis, cerebral haemorrhage in China since 1984 [60]. Interestingly, scutellarin was mainly absorbed by the intestine in the form of its hydrolyzed product scutellarein (Figure 6) [61]. Pharmacodynamics confirmed that scutellarein could prevent thrombosis and platelet aggregation, and improve the hemorheology characteristics of rats. Our docking studies of scutellarein with thrombin (2R2M) showed that the inhibitory activity could be improved, if a side chain is introduced at position 8 in the A ring by the Mannich reaction, so in 2012, we synthesized some Mannich bases of scutellarein [62] by electrophilic substitution at the C-8 position. In particular, morpholinyl aminomethylene substituted compound **27** (Figure 6), demonstrated stronger anticoagulant activity, and better water solubility compared with scutellarein.

In a program to systematically assess the chemical and biological diversity of seaweeds distributed in the Gulf of China’s Yellow Sea, Shi *et al.* [63] isolated a bromophenol derivative **28** (Figure 6) from the brown alga *Leathesia nana*, and they found that **28** was a potent thrombin inhibitor (IC_50_ = 1.03 nmol/L). Furthermore, the investigation of its antithrombotic efficacy *in vivo* using the arteriovenous shunt model and the ferric chloride-induced arterial thrombosis model in rats, indicated that **28** reduced the wet weight of the thrombus in a dose-dependent manner.

Cyanobacteria grow vigorously during algal blooms, release toxins, and cause eutrophication problems in lakes, reservoirs, and rivers [8]. Many cyanobacteria produce linear peptides that have protease inhibitory activities [64]. In 2012, Anas *et al.* isolated spumigin J (Figure 6) [65] with an *N*-methyllysine moiety, together with the known compound spumigin A (Figure 6) as thrombin inhibitors. The absolute configuration of spumigin J was analyzed by advanced Marfey’s methodology. Spumigin J and spumigin A inhibited thrombin with EC_50_ values were 4.9 and 2.1 μM respectively.

Radix Salviae Miltiorrhizae (RSM, “Danshen” in Chinese), the roots of *Salvia miltiorrhiza* Bunge (Labiate), is a traditional medicine commonly used in China for the treatment of cardiovascular diseases such as thrombosis [66]. Although RSM has perfect therapeutic effect on thrombosis in clinic, it is still not fully elucidated which components in RSM are responsible for its biological activities. In 2015, Lu *et al.* used a peak fractionation approach combined with an activity assay method [67] to screen direct thrombin inhibitors from RSM. A total of 91 fractions were collected from the RSM extract, and 19 fractions out of them showed thrombin inhibitory effects with dose-effect relationship. Among them, three compounds were unambiguously identified as 15,16-dihydrotanshinone I, cryptotanshinone and tanshinone IIA (Figure 6) with IC_50_ values were 29.39, 81.11 and 66.60 µM respectively.

## 3. Conclusions

After more than three decades of intensive research, significant progress has been made in the development of synthetic thrombin inhibitors as new anticoagulants, which is one of the most competitive areas in the field of medicinal chemistry. In order to fulfill the prerequisite for a convenient once or twice daily dosing regimen, research for the development of highly potent and selective inhibitors has paid more attention to their improved safety and a suitable half-life. Furthermore, several allosteric inhibitors have been designed and synthesized, which did not fully nullify the procoagulant signal, and thus could result in reduced bleeding complications. In the meantime, some new natural products with thrombin inhibitory activity have been isolated, and in order to improve their inhibitory activities and metabolic stability, some of these natural products have been modified. These new oral anticoagulants will create a new era in the management of venous and arterial thromboembolism, as they are more convenient because their oral route of administration, have lower bleeding rates, rapid action on- and offset, wide therapeutic windows, predictable dose responsiveness, and reduced interactions with food or other agents. All these efforts give a new hope and confidence to treat thrombotic disorders with selective and orally active thrombin inhibitors.
